# Glycobiology in osteoclast differentiation and function

**DOI:** 10.1038/s41413-023-00293-6

**Published:** 2023-10-26

**Authors:** Shufa Yang, Ziyi He, Tuo Wu, Shunlei Wang, Hui Dai

**Affiliations:** 1 Prenatal Diagnostic Center, Beijing Obstetrics and Gynecology Hospital, Capital Medical University, Beijing Maternal and Child Health Care Hospital, Beijing, 100026 China; 2 Department of Immunology, School of Basic Medical Sciences, NHC Key Laboratory of Medical Immunology, Peking University, Beijing, 100191 China

**Keywords:** Bone, Osteoporosis

## Abstract

Glycans, either alone or in complex with glycan-binding proteins, are essential structures that can regulate cell biology by mediating protein stability or receptor dimerization under physiological and pathological conditions. Certain glycans are ligands for lectins, which are carbohydrate-specific receptors. Bone is a complex tissue that provides mechanical support for muscles and joints, and the regulation of bone mass in mammals is governed by complex interplay between bone-forming cells, called osteoblasts, and bone-resorbing cells, called osteoclasts. Bone erosion occurs when bone resorption notably exceeds bone formation. Osteoclasts may be activated during cancer, leading to a range of symptoms, including bone pain, fracture, and spinal cord compression. Our understanding of the role of protein glycosylation in cells and tissues involved in osteoclastogenesis suggests that glycosylation-based treatments can be used in the management of diseases. The aims of this review are to clarify the process of bone resorption and investigate the signaling pathways mediated by glycosylation and their roles in osteoclast biology. Moreover, we aim to outline how the lessons learned about these approaches are paving the way for future glycobiology-focused therapeutics.

## Introduction

Glycobiology is the study of glycans and their recognition by motif-specific glycan-binding proteins or lectins. Glycans are ubiquitous in every cell and found on most secreted proteins. Glucocalyx, a dense layer of glycans on cell surfaces, can extend more than 30 nm from the plasma membrane. Glycosylation has a profound influence on protein activity and cell biology through a variety of mechanisms. Furthermore, dysregulated glycosylation plays a crucial role in disease processes, ranging from immune evasion to diminished cognition, spurring research on the therapeutic use of glycans. Glycobiology research is key to providing a new perspective on understanding the pathogenesis of disease.

Bone protects vital organs and acts as a mineral reservoir that is essential for calcium homeostasis. The stability of bone mass is maintained by the dynamic balance between bone resorption by osteoclasts and bone formation by osteoblasts. Increased osteoclast (OC) differentiation and activity are critical and result in bone loss and joint destruction in common pathological bone conditions, such as osteoporosis and rheumatoid arthritis (RA). Osteoclasts are multinucleated cells of monocyte/macrophage origin that degrade bone matrix. The differentiation of osteoclasts depends on nuclear factor (NF)-kappaB ligand (RANKL) and macrophage colony-stimulating factor (M-CSF). The process is physiologically guided by a diverse set of hormones, cytokines and growth factors.

Here, we summarize the role of glycosylation in diseases involving the bone and joint system. Since osteoclasts play a key role in the process of rheumatoid arthritis, osteoarthritis, tumor bone metastasis and bone erosion, we focused on the effects of various glycan structures and glycosylation processes on osteoclastogenesis.

## Glycosylation process

### Introduction to glycosylation

Glycosylation mainly occurs in the endoplasmic reticulum and Golgi. Glycoprotein synthesis is coordinated by the sequential action of glycosyltransferases (GTs) and glycoside hydrolases (GHs). The process includes an initiation step, extension of the sugar core, an extension/branching step and a capping step. The core sugar initiation step and elongation of each type of protein glycosylation vary and are regulated by one or more specific glycosyltransferases.^1–3^

### N-glycosylation initiation and core extension

**Fig. 1:**
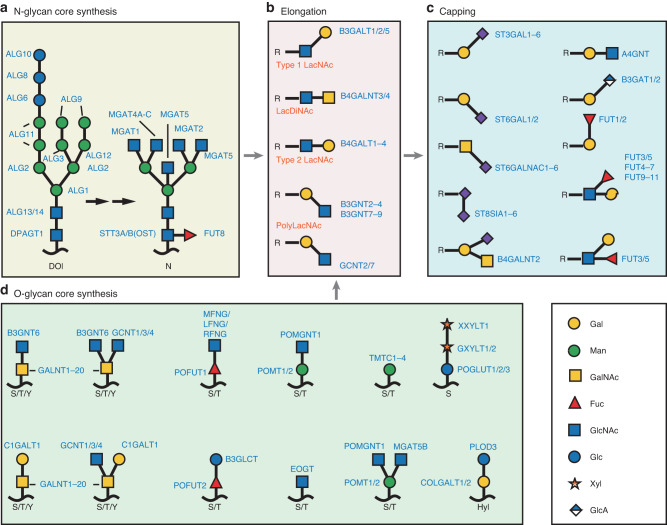
Schematic Illustration of Human Glycoprotein Modification. The primary glycosylation pathways are categorized based on the initiation, core extension/branching, elongation and branching, and capping steps. Glycosyltransferases are indicated in proximity to the transferred monosaccharides. **a** Initiation and core elongation of N-glycosylation: After prior synthesis of precursors on DOI, they are transferred to N residues as catalyzed by STT3A/B (OST). **b** Elongation and branching stages of protein glycosylation. **c** Capping steps in protein glycosylation. **d** Initiation and core elongation of O-glycosylation. R: variable core glycan. Dol dolichol. N Asn. S: Ser. T Thr. Y Tyr. OST oligosaccharyl transferase

N-glycans are linked by N-glycosidic bonds formed between the reducing end of GlcNAc and the amide nitrogen atoms of asparagine (Asn) residues within the sequence located within the Asn-Xaa-Ser/Thr sequence protein fragment, where Xaa represents proline. In the synthesis of N-glycans, the first step involves the formation of polyhydroxypyrophosphate (Dol-PP)-linked high mannose precursor oligosaccharides. Subsequently, oligosaccharide precursors are “bulk” transferred from Dol to a protein by the oligosaccharyltransferase (OST) complex.^4^ After the overall translocation of the precursor oligosaccharide into protein, the glucose and mannose residues are sequentially removed by glucosidase I and II. Subsequently, N-glycoprotein undergoes reglycosylation under the direction of different glycosyltransferases^5,6^ (Fig. 1a).

### O-Glycosylation initiation and core extension

O-linked glycans within glycoproteins are formed because of the relationship between a hydroxyl group on the sugar isomer hydroxyl moiety and the hydroxyl group of serine (Ser), threonine (Thr), tyrosine (Tyr) or hydroxylysine (Hyl) residues. O-glycosidic bonds establish glycan-modified proteins. O-linked glycans are transferred to synthesized proteins via specific glycosyltransferases (Fig. 1d). In one classification, O-linked glycans are categorized on the basis of the initial monosaccharide attached to the protein. Fuc, Glc, Man and GlcNAc O-glycosylation is initiated in the endoplasmic reticulum (ER). The Golgi apparatus initiates the synthesis of GalNAc-type O-glycosylation on Ser/Thr/Tyr residues.

The GalNAc transferase (GALNT) isozyme initiates the synthesis of GalNAc-type O-glycosylation on Ser/Thr/Tyr residues, which extends the core structure through the action of B3GNT6, GCNT1/3/4, and C1GALT1.^7–10^ POFUT1/POFUT2 promote the initiation of Fuc-type O-glycosylation. The extension of the Fuc-type O-glycosylated core structure is catalyzed by B3GLCT/MFNG/LFNG/RFNG.^11–13^ Man-type O-glycosylation is initiated by POMT1/2 and TMTC1-4, and its core extension is catalyzed by POMGNT1 and MGAT5B.^14–18^ POGLUT1/2/3, EOGT and COLGALT1/2 mediate the initial synthesis of Glc-type, GlcNAc-type and Gal-type O-glycosylation, respectively.^14–17^ The extension of the Glc-type O-glycosylation core is achieved through the action of XXYLT1 and GXYLT1/2.^18–20^ PLOD3 is involved in the elongation of the Gal-type O-glycosylation core.^21^

### Unspecific steps of the glycosylation process

The initiation, core branching, and elongation steps of various aforementioned protein glycosylation types are specific to each type. The subsequent process of glycan elongation, branching and final capping is nonspecific, and some enzymes are shared among different types of glycoprotein and even glycolipid synthesis pathways.

Type 1 LacNAc (Galβ1–3GlcNAc), Type 2 LacNAc (Galβ1–4GlcNAc), LacDiNAc (GalNAcβ1–4GlcNAc) and repeating disaccharides (polyLacNAc) are involved in the elongation and branching of the core glycan structure. Elongation and branching of the glycan core involve B3GALT1-9, B4GALNT1-4, and GCNT2/7^10,22–24^ (Fig. 1b).

The capping step mainly involves the terminal modification of the oligosaccharide chain. Neu5Ac, Fuc, GlcNAc and GalNAc are transferred to the end of a oligosaccharide chain via the action of a glycosyltransferase. The glycosyltransferases involved in the capping step include FUT, ST3GAL, ST6GAL, ST6GALNAC, ST8SIA, B3GAT, and A4GNT^25–29^ (Fig. 1c).

## The function of glycosylation in osteoclastogenesis and the underlying mechanism

### Sialic acid glycosylation

Sialic acid is a negatively charged monosaccharide; the two most common classes of sialic acids found in animals are N-acetylneuraminic acid (Neu5Ac) and N-glycolylneuraminic acid (Neu5Gc). Humans lack the ability to synthesize Neu5Gc because of a prevalent inactivating mutation in the gene encoding CMP-N-acetylneuraminic acid hydroxylase; therefore, the predominant sialic acid in humans is N-acetylneuraminic acid. Sialic acids are often attached to the termini of cell surface glycan chains. Sialic acid-containing glycans are key components of the glycocalyx. Previous studies reported that sialic acid functions primarily to provide a negative charge and hydrophilicity to cell surfaces, mask subterminal galactose residues recognized by certain receptors, and act as receptors for pathogens and toxins. Sialic acid plays an important role in cell‒cell interactions and cell signaling.^30^

Early studies of the lectin histochemistry of pathological bone tissue revealed both membrane and cytoplasmic distribution of sialic acid in osteoclasts and osteoclast precursor cells.^31^ Later studies demonstrated the presence of α (2,3)-linked sialic acids and α (2,6)-linked sialic acids in mouse bone marrow-derived macrophages and the RAW264.7 macrophage line. Notably, α(2,3)-linked sialic acids are abundant throughout osteoclastogenesis, whereas α(2,6)-linked sialic acid levels are decreased at the end of osteoclast differentiation. This suggests that α (2,6)-linked sialic acids may play roles in the process of osteoclast formation.^32^

The formation of osteoclasts was inhibited in RAW264.7 macrophages when the α (2,6)-linked sialic acid level was reduced by glucosamine treatment.^33^ Osteoclasts differentiate from hematopoietic precursors of monocyte/macrophage lines and form multinucleated giant cells through cell‒cell fusion. The process of multinucleation is considered a key step in osteoclast formation and functional differentiation.^34^ Notably, desialification inhibited the formation of TRAP^+^ multinucleated giant cells and osteoclast differentiation induced by M-CSF and RANKL in a dose-dependent manner.^32^

One possible mechanism by which sialylation plays a role in osteoclast fusion is the binding of sialylated TLR2 on osteoclast precursor cells to siglec-15, which induces cell‒cell fusion and promotes RANKL-induced osteoclastogenesis.^35^ Inhibition of sialylation in RANK^+^ TLR2^+^ monocytes impeded osteoclast fusion, and reduced expression of the fusion-associated genes *Cd44*, *Cd47* and *Ocstamp* was detected.^36^

Furthermore, sialic acid plays an important role in the interaction between osteoclasts and other cells. It is widely involved in biofilm recognition, fusion, and receptor‒ligand interactions. As a tumor cell line capable of bone metastasis, the PC3 prostate cancer cell line^37^ can generate tumor-derived extracellular vesicles (PC3-EVs) to promote osteoclast activity and bone metastasis. However, the endocytosis of vesicles by RAW264.7 cells can be significantly inhibited by the ceramidase-mediated removal of sialic acid from the PC3-EV surface, indicating a key role of sialic acid in membrane recognition and fusion.^38^ The DCIR (Dendritic cell immunoreceptor) receptor expressed by dendritic cells (DCs) or osteoclasts can bind to NA2 (Asialo-biantennary-N-glycans) polysaccharide molecules on the surface of osteoclasts to inhibit osteoclast activity. The removal of sialic acid from the surface of NA2 polysaccharide molecules by ceramidase can significantly increase the exposure of NA2 and enhance the inhibitory effect on osteoclast activity. Notably, sialic acid plays a role in the most traditional sense; that is, it exerts a masking effect on medial galactose residues.^39^ Sialic acid can be regarded as a potential therapeutic target for the suppression of tumor bone metastasis and the treatment of autoimmune osteoarthropathy.

However, another study showed a contrasting result. After adding sialic acids to preosteoclasts, reduced bone resorption was observed. One possible mechanism involves sialic acid binding to siglec-9 on osteoclast precursors through a tyrosine motif, resulting in IL-10 secretion.^40^ The opposite results obtained indicate that we lack a clear understanding of the molecular and cellular mechanisms underlying sialic acid effects on osteoclastogenesis.^41^

### O-GlcNAc

O-GlcNAcylation is transient, inducible, reversible and dynamic posttranslational glycosylation. The modified structure is a single hexose molecule.^42^ O-GlcNAcylation and phosphorylation enzymes modify the same sites, namely, the serine and threonine hydroxyl groups on a target protein. A dynamic balance is maintained between these modifications to coordinate a variety of complex life activities. Any imbalance in these modifications leads to disease. O-GlcNAcylation regulation is catalyzed by two enzymes, OGT and OGA.^43^ OGT promotes O-GlcNAcylation, and OGA promotes deglycosylation. OGT is highly sensitive to the concentration of its substrate UDP-GlcNAc, which is produced via the aminohexose synthesis route of the glucose metabolism pathway. In summary, glucose levels affect the glycosylation process, which indicates that O-GlcNAcylation is related to glucose metabolism and diabetes.^44^

O-GlcNAcylation in macrophages and osteoclasts directly inhibits the formation of osteoclasts. The number and activity of osteoclasts were significantly reduced after the rate of O-GlcNAcylation was increased using GlcN, GlcNAc, and PUGNAc (an OGA inhibitor).^33,45–49^

One of the main mechanisms involves an increase in O-GlcNAcylation that inhibits the phosphorylation of signaling pathway molecules downstream of RANKL, especially p65 and IκBα, resulting in reduced osteoclastogenesis.^33,45^ During later stages of osteoclastogenesis, phosphorylation of signaling pathways is inhibited, resulting in decreased cell fusion, increased actin cytoskeletal rearrangement, and integrin-mediated cell adhesion-mediated inhibition of osteoclast development.^46^

Another possible mechanism involves the O-GlcNAcylation regulation of the entry of transcription factors into the nucleus. O-GlcNAc acylation of the transcription factors p65 and NFATc-1 is required for their nuclear translocation.^47,48^ The O-GlcNAcylation of the nucleoporin NUP153 is significantly upregulated during osteoclastogenesis^46^ and has been shown to promote the nuclear translocation of MYC, thereby increasing the expression of osteoclast genes.^46,49^

Additionally, O-GlcNAcylation may affect osteoclast formation by affecting other glycosylation processes. For example, UDP-GlcNAc is the substrate of O-GlcNAcylation; however, it can also be converted into CMP-Neu5AC, which is the substrate of sialic acid glycosylation.^50^ Studies have shown that increased O-GlcNAcylation reduces the amount of α2,6-linked sialic acid-modified glycoproteins on the surface of osteoclast precursors.^33^ This is an important finding, as α2,6-linked sialic acid modification is important for the fusion of osteoclast precursors. After removing α2,6-linked sialic acid with SAase, osteoclast formation was significantly reduced.^32^

### Antibody glycosylation

IgG binds to antigen via the Fab segment and to complement or Fc gamma receptor (FcγR) via its Fc segment. Glycosylation of antibodies occurs mainly on the Asn-297 residue of Fc in the form of N-glycosylation.^51^ This modification increases the diversity of antibodies and influences their affinity for the receptor FcR, affecting the functions of antibodies such as antibody-dependent cytotoxicity (ADCC), complement-dependent cytotoxicity (CDC), and antibody-dependent cytophagocytosis (ADCP).^52^

**Fig. 2:**
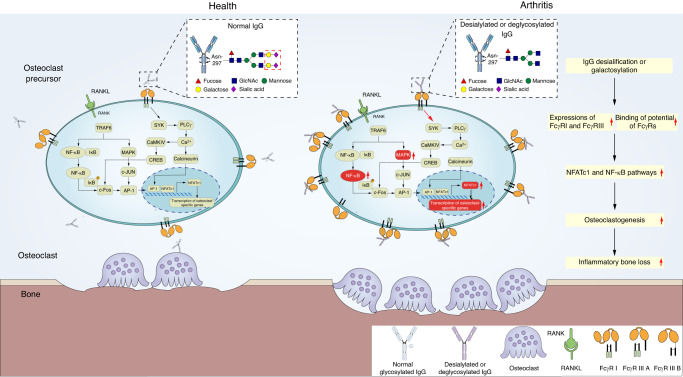
Desialification or degalactosylation of antibodies induces inflammatory bone loss by increasing osteoclastogenesis. A high degree of sialic acid on IgG Fc can counteract the binding potential of FcγRs and reduce the affinity of IgG for FcγR, thus inhibiting downstream effects and leading to less inflammation (left). Desialification or degalactosylation of antibodies increases the expression of FcγRI and FcγRIII and the binding potential of FcγRs, which then significantly activate the NFATc1 and NF-kB pathways to promote osteoclastogenesis and inflammatory bone loss (right)

T.T. Wang et al. systematically reviewed the effect of Fc glycosylation on the functional diversity of IgGs.^51^ Modification of the core IgG Fc glycan at the conserved Asn297 site can regulate the structure of the Fc segment, determine the type of FcγR to which it binds, and affect its affinity for FcγR. The presence of core fucose on the Fc glycan moiety reduces IgG affinity for FcγR IIIA.^53^ Site-selective modification of core fucose enhances IgG affinity for FcγR.^54^ A high degree of sialic acid on the IgG Fc reduces the affinity of IgG for FcγRs, thus inhibiting downstream effects and attenuating inflammation. In contrast, IgG lacking terminal sialic acid shows increased affinity for FcγR and therefore exerts proinflammatory effects^55^ (Fig. 2).

The galactose and sialic acid contents on IgG from patients with joint diseases are significantly reduced compared with those on healthy individuals.^56^ The degree of reduction is significantly correlated with the rate of decrease in bone volume and trabecular number and the rate of increase in level of serum CTX-1 (a marker of bone resorption).^57,58^ After desialification or degalactosylation, the osteoclast generation rate is increased and is accompanied by NFATc1 and NF-κB pathway activation.^55,59^ Furthermore, increased expression of FcγRI and FcγRIII has been reported, highlighting the importance of sialic acid.^55,57,58^ In addition, IgG that is glycosylated at a low level induces bone loss in healthy joints but does not significantly aggravate existing arthritis. Modulating the rate of antibody glycosylation can ameliorate the symptoms of joint inflammation. For example, phytoestrogen administration that increases IgG glycosylation can relieve inflammation and inhibit the NF-κB pathway and NFATc1/c-fos axis to decrease the activity of osteoclasts, protecting mice from collagen-induced arthritis (CIA).^59^ Treatment with the sialic acid precursor N-acetylmannosamine (ManNAc) to increase IgG sialylation can reduce inflammatory-induced bone loss.^58–60^ For patients with multiple myeloma, tumor load and bone loss can be reduced by modifying the IgG glycosylation rate.^57^

## Free glycans

### Proteoglycans and glycosaminoglycans

Glycosaminoglycan is a negatively charged long-chain linear heteropolysaccharide composed of repeating disaccharide structures. Proteoglycan (PG) is covalently linked by one or more glycosaminoglycans (GAGs) and core proteins. It can be modified by sulfate groups at different positions. Glycosaminoglycans include CS, dermatan sulfate (DS), keratin sulfate (KS), hyaluronic acid (HA), heparin, and heparan sulfate.^61,62^ Proteoglycan is the main component of the extracellular matrix. Its biological function is mainly based on the physical and chemical properties of glycosaminoglycan, and affects various cell processes, including growth and migration.

#### Hyaluronic acid (HA)

Hyaluronic acid (HA) is composed of D-glucuronic acid and N-acetyl-D-glucosamine linked by alternating β-(1 → 4) and β-(1 → 3) glycosidic bonds. The activity of hyaluronidase is increased in the peripheral blood of patients with RA and can be used as an inflammatory marker.^63–66^ Hyaluronidase significantly reduces the level of HA in the synovial fluid of patients with RA.^67^ However, according to the consensus statement of relevant professional societies, hyaluronic acid injections are generally not recommended for the treatment of RA or OA.^68–71^

The current consensus is that HA inhibits RANKL-induced osteoclastogenesis and directly reduces the function of mature osteoclasts.^72^ Possible mechanisms underlying these outcomes include decreased RANKL expression or binding with CD44 or TLR4. High-molecular-weight HA (>90 kD) inhibits RANKL expression in bone marrow stromal cells by activating the RhoA/Rho kinase pathway^73^ or by regulating the phosphorylation of VDR and STAT3.^74^

HA of different molecular weights can regulate the process of osteoclast differentiation and formation by interacting with the Type I transmembrane glycoprotein CD44, a common receptor for HA.^73,75^ Binding of HA to CD44 downregulates NFATc1 activity, leading to decreased expression and activity of MMP-9, cathepsin K and TRAcP, thereby impairing the resorptive capacity of osteoclasts.^76^ Moreover, HA can inhibit the migration and adhesion of osteoclasts by reducing the expression of integrin β3 and carbonic anhydrase II induced by RANKL.^72^ Owing to its high affinity for CD44, HA is widely used as a targeted carrier for the treatment of osteoarthritis, RA, and osteoporosis.^77–79^ However, a study found that hyaluronic acid administered in vitro during the differentiation of osteoclast precursors still inhibited RANKL-induced osteoclastogenesis in CD44KO mice.^80^

**Fig. 3:**
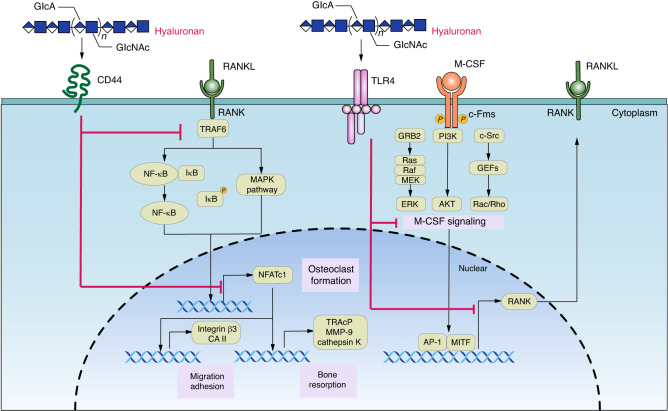
Hyaluronan (HA) regulates signaling pathways in osteoclast precursor cells. Hyaluronan (HA) is a linear polysaccharide consisting of disaccharide units, which are D-glucuronic acid and N-acetyl-D-glucosamine. HA downregulates NFATc1 signaling pathways via interaction with CD44 and impairs the resorption activity of osteoclasts by decreasing MMP-9, cathepsin K, and TRACP expression. Furthermore, it inhibits osteoclast migration and adhesion by decreasing RANKL-induced integrin β3 and carbonic anhydrase II expression. High-molecular-weight HA also interferes with M-CSF signaling in osteoclast precursors in a TLR4 receptor-dependent manner, resulting in decreased activation of the transcription factors AP-1 and MITF and decreased expression of RANK, inhibiting osteoclast differentiation

In addition, high-molecular-weight HA suppressed M-CSF signaling in osteoclast precursors in a TLR4 receptor-dependent manner, resulting in decreased activation of the transcription factors AP-1 and MITF and decreased expression of RANK, thereby inhibiting osteoclast differentiation^81^ (Fig. 3). A study showed that LPS also activated the TLR4 signaling pathway to reduce the expression of c-Fms on bone marrow-derived macrophages.^82^ It is not clear whether the binding of HA and TLR4 causes similar changes in osteoclast precursor cells to regulate M-CSF signaling.

Notably, the effect of HA on osteoclastogenesis remains controversial. It has been reported that low-molecular-weight HA (8 kD) can promote the phosphorylation of ERK and p38 MAPK signaling pathways and c-Src expression in osteoclast precursor cells to increase the osteoclast formation rate.^75^ On the one hand, sulfated HA can inhibit the formation of osteoclasts; on the other hand, it can competitively bind OPG, a decoy RANKL receptor, to promote osteoclastogenesis.^83,84^

#### Chondroitin sulfate (CS)

Chondroitin sulfate (CS) is a long-chain polysaccharide consisting of a repeating disaccharide structure composed of N-acetylgalactosamine and glucuronic acid. Most acetylgalactosamine residues are sulfated, resulting in CS structural and functional diversity. Based on the sulfation status of repeating disaccharide units, CS can be classified into four categories, namely, chondroitin sulfate-4 (chondroitin sulfate A), chondroitin sulfate-6 (chondroitin sulfate C), chondroitin sulfate-2,6 (chondroitin sulfate D) and chondroitin sulfate-4,6 (chondroitin sulfate E). Dermatan sulfate, also known as chondroitin sulfate B, shares structural and functional characteristics with chondroitin sulfate.^85^ Chondroitin sulfate significantly inhibits the formation, differentiation and various functions of osteoclasts.^84,86,87^ Chondroitin sulfate E inhibits osteoactivin-induced osteoclast differentiation by blocking the interactions of osteoactivin with integrin αVβ3 and HSPG;^88^ dermatan sulfate binds to RANKL to inhibit osteoclast differentiation.^89^ It participates in the synthesis and metabolic balance of the extracellular matrix. Moreover, it has potential anti-inflammatory activity and has been used to treat osteoarthritis.^90^

#### Heparin

The effects of heparin on bone metabolism and osteoclastogenesis have been widely explored. Early studies showed that heparin can cause cancellous bone loss in rats by increasing the number and activity of osteoclasts.^91^ However, heparin also has a biphasic effect on osteoclast formation. Folwarczna et al. reported that heparin tends to increase osteoclast formation at low concentrations while reducing the number of osteoclasts at high concentrations.^92^

**Fig. 4:**
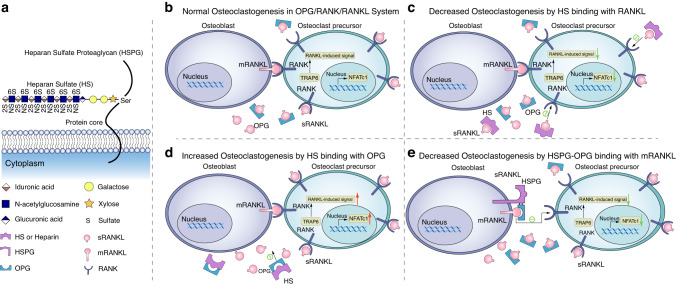
Effect of heparin and heparan sulfate on the OPG/RANKL/RANK system in the regulation of osteoblasts and osteoclasts. **a** Heparan sulfate (HS) is a polymer of D-glucuronic acid or L-iduronic acid and D-glucosamine (GlcA) sugars. **b** The OPG/RANK/RANKL regulatory system between osteoblasts and osteoclasts under normal conditions. **c** Heparin binds with sRANKL to decrease sRANKL binding to RANK and inhibits the downstream signal of RANK to reduce osteoclastogenesis. **d** Heparin binds to OPG, which competitively inhibits the binding of OPG to the RANK-RANKL complex, promotes osteoclast formation and increases osteoclast absorption activity. **e** OPG is fixed to HSPG on the surface of osteoblasts after binding to HS, which increases the probability of successful binding between OPG and RANKL and increases the efficiency of OPG inhibiting RANKL downstream signaling. Heparan sulfate (HS); heparan sulfate proteoglycan (HSPG)

Heparin can affect osteoclast formation directly or indirectly by binding to either RANKL or OPG. Heparin increased the expression of gp130 and RANKL in osteoblasts, promoted the formation of osteoclasts in a calvarial and bone marrow cell coculture system, and increased the IL-11-induced nonphosphorylated activation of STAT3 by upregulating the MAPK signaling pathway.^93,94^ Heparin also binds to OPG and competitively inhibits the binding of OPG to RANKL to promote osteoclast formation and increase osteoclast absorption.^95,96^ When heparin binds to RANKL, downstream RANKL signaling is inhibited, which reduces the adhesion ability of osteoclast precursors or inhibits the proliferation of osteoclasts.^97,98^ (Fig. 4)

#### Heparan sulfate (HS)

Heparan sulfate (HS) is a highly sulfated glycosaminoglycan composed of repeated trisulfated disaccharide units composed of L-iduronic acid and D-glucosamine. HS is linked to a core protein to form heparan sulfate proteoglycan (HSPG), which mediates numerous biological processes, including cell growth, adhesion, and migration and angiogenesis.

The effect of HS chain elongation on osteoclastogenesis has been well documented. The *EXT1/2* gene encodes glycosyltransferases necessary for HS chain elongation. A significant increase in the number of osteoclasts was found in *Ext1*^−/−^ mice. *EXT1/2* mutations have also been shown to be associated with hereditary multiple osteochondroma (MHE), an inherited form of bone dysplasia characterized by short stature, multiple cartilage-covered tumors throughout the body, and decreased bone density.^99,100^ SLC10A7 mutations significantly reduce heparan sulfate levels in mouse chondrocytes and patient fibroblasts. Hypomineralization and hyperameloblasts were found in patients with SLC10A7 deletion presenting with skeletal dysplasia with multiple dislocations.^101^ Loss of TMEM165 impairs heparan sulfate chain elongation, resulting in a skeletal disorder characterized by severe skeletal dysplasia and overt dwarfism.^102^

OPG can bind to HS to inhibit RANKL/RANK signaling and osteoclastogenesis.^103^ Although OPG is sometimes immobilized on HSPG on the surface of osteoblasts, the successful binding of OPG to RANKL is more likely and is followed by a decrease in RANKL signaling.^104,105^

Heparan sulfate in the extracellular domain of syndecans can bind with M-CSF and block M-CSF-mediated downstream signaling, including ERK, cJNK, p38 and Akt signaling, thereby inhibiting osteoclastogenesis.^106^ The receptor complex Syndecan-1/M-CSFR is essential for myeloid IL-34/M-CSFR signaling and downstream functions. IL-34-induced osteoclast formation and joint inflammation were significantly reduced in syndecan-1-deficient mice.^107^ Li et al. found that NF-κB activation promoted syndecan-4 transcription, which induced osteoclast differentiation by enhancing RANKL-induced autophagy.^108^

HS has also been shown to bind directly to a variety of factors related to skeletal development, including but not limited to BMP, TGF-β, FGF, and Wnt.^109,110^ FGF-2 produced by synovial fibroblasts in the RA context can be transferred to FGFR-1 by binding to HSPG, leading to RANKL- and ICAM-1-mediated osteoclast activation and maturation, resulting in bone destruction.^111^

### Natural products

**Fig. 5:**
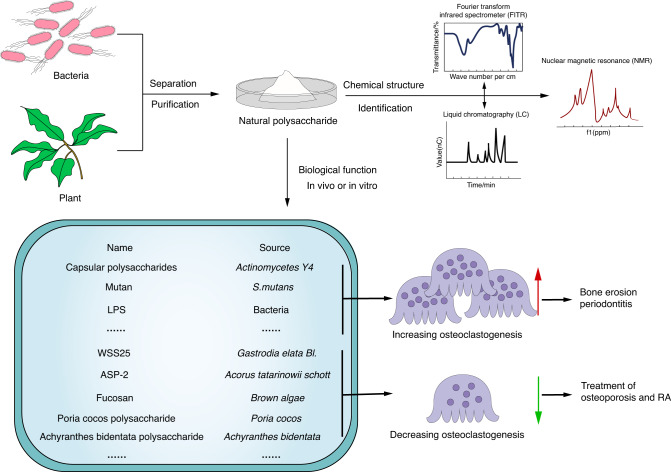
Extraction, characterization and biological functions of natural polysaccharides. Natural polysaccharides can be separated and purified from plants, animals and microorganisms. Following identification of the chemical structure via mass spectrometry and nuclear magnetic resonance, the pharmacological activities of natural polysaccharides were detected in vivo and in vitro. Some polysaccharides extracted from *actinomycetes Y4, S. mutans*, and some bacteria have been proven to increase osteoclastogenesis, while others, derived from *Gastrodia elata*, *Acorus tatarinowii Schott*, *brown algae*, etc., have been reported to inhibit osteoclastogenesis

Natural polysaccharides are widely distributed in plants, animals and microorganisms. Due to their wide pharmacological activities, such as antitumor, immunomodulatory, and anti-inflammatory effects, they have gained increasing attention^112^ (Fig. 5). Studies have shown that capsular polysaccharides purified from *actinomycetes Y4* induced osteoclast formation and bone resorption. The use of IL-1α-specific monoclonal antibody completely blocked this promoting effect, suggesting that IL-1α is involved in the induction of capsular polysaccharides on osteoclasts.^113^ Mutan is an extracellular α-(1,3)(1,6)-D-glucan from *S. mutans* that promotes osteoclast differentiation and bone mass loss, suggesting that it may be involved in inflammatory responses in periodontal disease.^114^ Numerous reports have shown that LPS in gram-negative bacteria is important for osteoclastogenesis.^115–117^ LPS can act by binding to the TLR4 receptor and affecting downstream molecules, including TNF-α and CXCR4, thereby promoting osteoclastogenesis.^118,119^ Additionally, drugs including artesunate and sinsinine can inhibit the induction of osteoclasts by LPS by inhibiting the TLR4/TRAF6 signaling pathway.^120,121^ It is worth noting that LPS acts differently on osteoclastogenesis in vivo and in vitro. LPS plays an important role in promoting osteoclastogenesis in vivo, which requires the help of the microenvironment. LPS stimulates immune cells to secrete proinflammatory cytokines such as IL-6, TNF-α, and IL-1,^122–124^ and these cytokines can stimulate osteoblasts to secrete RANKL to promote the differentiation and activation of osteoclasts.^125–127^ In contrast, when LPS was added directly to in vitro OC cultures, the differentiation of untreated osteoclast precursor cells was significantly inhibited.^128,129^

A range of polysaccharides extracted from plants and other natural medicines have shown inhibitory effects on osteoclasts and good anti-osteoporotic potential. WSS25 is a sulfurated derivative of α-1-4-linked glucan extracted from the rhizome of *Gastrodia elata BI*.WSS25. It inhibits RANKL-induced osteoclast formation from RAW264.7 cells and bone marrow-derived macrophages (BMMs) by blocking the BMP-2/Smad/Id1 signaling pathway.^130^ The polysaccharide ASP-2 derived from the root of *Acorus tatarinowii Schott* inhibits osteoclast differentiation and attenuates LPS-induced bone loss in mice by inhibiting the PLCγ2-Ca^2+^ signaling axis.^131^ Fucosan derived from *brown algae* inhibits intracellular Ca^2+^ signaling and calcitonin activity by regulating the Akt/GSK3β/PTEN signaling pathway, thereby inhibiting the intracellular migration of NFATc1 to the nucleus, thereby inhibiting osteoclast activation and bone resorption. This inhibitory effect can be reversed by kemparodone, a GSK3β inhibitor.^132^ Song et al. reported that *Poria cocos*, *Achyranthes bidentata*, and *Cistanche* polysaccharides inhibited osteoclastogenesis and bone resorption by inhibiting the RANKL signaling pathway.^133–135^ Moreover, polysaccharides in some foods have been shown to have anti-osteoclast effects. For example, polysaccharide porphyrins derived from edible seaweed inhibited the formation of osteoclasts induced by RANKL,^136^ and tea polysaccharides inhibited the differentiation of osteoclast progeny into osteoclasts in a dose-dependent manner.^137^ In addition, in rat models, both tea and *Cuscuta* polysaccharides were useful for attenuating postmenopausal osteoporosis.^137,138^ In contrast, the oral administration of stem polysaccharides derived from *Dendrobium officinale Huosanum* effectively alleviated joint swelling, synovial hyperplasia and bone destruction in CIA mice. Moreover, it regulated the balance of Th17/Treg cells; reduced the activation of fibroblast-like synovial cells, angiogenesis and the secretion of proinflammatory mediators; and showed the potential to treat rheumatoid arthritis.^139^

### Advanced glycation end products (AGEs)

Although the mechanism has not been fully clarified, advanced glycation end products (AGEs) play key roles to increase the risk of fracture in diabetic patients, and it has been shown that AGEs can inhibit the activation and induce the apoptosis of osteoblasts. However, its role in osteoclasts remains controversial. Early studies showed that AGEs enhanced the bone resorption activity of osteoclasts.^140,141^ However, recent studies showed that AGEs significantly inhibited osteoclast differentiation and formation. For example, Park et al. found that treatment of bone marrow-derived macrophages with AGEs resulted in a significant reduction in Trap^+^ multinucleated giant cell formation and downregulation of osteoclast-specific gene expression,^142^ and Tanaka et al. similarly reported that AGEs modified by glycolaldehyde inhibited osteoclast differentiation by influencing the expression of IL-10 induced by NF-κB.^143^ These contrasting results may be partially explained by the different effects of AGEs in different biological stages of osteoclasts. AGEs inhibit osteoclast generation by inhibiting RANK expression in osteoclast precursors during osteoclast formation, while they increase bone resorption capacity by increasing the number of podosomes during osteoclast maturation.^144^ In addition, another study focused on the role of the AGE receptor RAGE in osteoclasts: AGEs and age-related proteins interact with RAGE to affect osteoclasts.^145,146^ A *RAGE*^*−/−*^ mouse model confirmed that defects in RAGE can lead to an increase in bone mass and bone mineral density and a decrease in the number and functional impairment of osteoclasts.^147,148^

Diabetes mellitus is often associated with an increased risk of osteoporosis and fragility fractures, possibly due to hyperglycemia, the accumulation of AGEs, and decreased serum levels of osteocalcin and parathyroid hormone.^149,150^ Insulin can promote bone anabolism, leading to more severe bone loss in patient with Type I diabetes patients than patients with Type II diabetes. Patients with Type II diabetes often present with altered bone microstructure and mechanical properties and accumulation of microdamage due to collagen fiber cross-linking caused by concurrent hyperinsulinemia and AGE accumulation.^151,152^

## Glycan-binding proteins in osteoclasts

### Sialic acid-binding immunoglobulin-like lectin (Siglec)

**Fig. 6:**
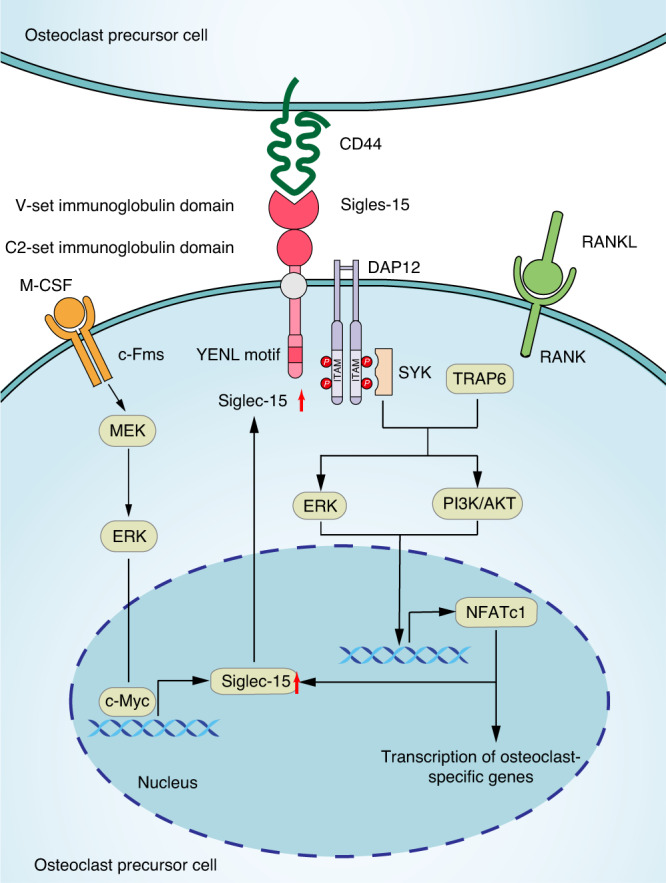
Signaling pathway of siglec-15 in osteoclast precursor cells. Siglec-15 contains two Ig-like domains, including a V-set-like domain (IgV) and a C2 set-like domain (IgC2). CD44 binds to Siglec-15 and activates the ITAM of DAP-12, recruits Syk, and promotes the phosphorylation of PI3K, Akt, and ERK to promote the formation of osteoclasts. M-CSF and NFATc1 can promote the expression of siglec-15

Siglecs are immunoglobulin-like lectins expressed on cell membranes that bind to sialic acid. Most siglecs have immunoreceptor tyrosine-based inhibitory motifs (ITIMs) that can recruit Src homology 2 domain-containing protein tyrosine phosphatase 1 (SHP-1) and transmit inhibitory signals.^153^ Siglec-15 is different from most lectins because it does not contain an ITIM but carries positively charged lysine residues that can combine with the DAP12 immunoreceptor tyrosine-based activation motif (ITAM) to transmit activation signals and promote osteoclast differentiation. (Fig. 6)

Siglec-15 is a type I transmembrane protein. Its extracellular domain contains two Ig-like domains, including a V-set-like domain (IgV) and a C2 set-like domain (IgC2). Between these domains, the IgV domain contains a conservative arginine motif that is particularly important for its binding with the ligand sialic acid.^154^ The transmembrane region contains lysine residues that bind to DAP12.^155^ There is no ITIM in the cytoplasmic region; however, there is a recognized endocytosis motif, YENL, that conforms to the universal endocytosis motif YxxØ (Ø represents a amino acid residue with large hydrophobic side chains) and mediates the endocytosis of Siglec-15 after the formation of actin ring.^156,157^

Siglec-15 promotes osteoclast differentiation and plays an important role in cell fusion and cytoskeleton formation. Siglec-15 was directly involved in pathological bone erosion in a K/BxN mouse model of serum transfer arthritis.^158^ In *Siglec-15* knockout mice, bone trabecular bone mass and bone density increased, and urine DPD (representative of bone absorption level) and TRAP activity decreased.^159,160^ Female *siglec-15* knockout mice are resistant to ovariectomy-induced osteoporosis,^161^ suggesting that loss of siglec-15 resulted in impaired osteoclast function. Shimizu et al. reported that in adjuvant-induced arthritic mice (AIA), siglec-15 only mediated periarticular bone loss and did not contribute to joint destruction.^162^ However, osteoclastogenesis pathway activation increases the expression of siglec-15 via a positive feedback loop. M-CSF leads to phosphorylation of Myc at Ser62 through the MEK-ERK-Myc signaling pathway, thereby activating giglec-15 expression.^35^ NFATc1, the core molecule of osteoclast differentiation, promotes the expression of siglec-15.^163^

Although the ligands for siglec-15 have not been specifically identified, the known ligands for siglec-15 include α-2,3 sialic acid,^35^ Neu5Acα2-6GalNAc (sTN)^157^ and CD44.^164^ Coimmunoprecipitation assays confirmed that CD44 and siglec-15 bind to each other and that cell fusion is attenuated in CD44-knockout osteoclasts.^164^ Siglec-15 can also bind to α-2,3 sialic acid-modified TLR2 and recruit MyD88 to bind and upregulate NFATc1 and p65 to promote osteoclast differentiation.^35^ Siglec-15 that had lost its glycan binding domain V-set was unable to rescue the function of *siglec-15*^*−/−*^ cells, demonstrating that binding to glycans via the V-set domain is required for siglec-15 to function.^163^

For siglec-15 to promote osteoclast activity, it needs to bind to DAP12^165^ or TLR2.^35^ After binding with DAP12, siglec-15 activates ITAM motifs and recruits Syk to promote the phosphorylation of PI3K, Akt, and ERK, which play important roles in osteoclast fusion, actin ring formation, and bone resorption. Under Siglec-15 deficiency conditions, the phosphorylation of PI3K, Akt, and ERK was inhibited.^165^ Normal exogenous siglec-15 was able to prevent the death of siglec-15-deficient mice, but when it failed to bind DAP12, siglec-15 did not rescue the mice, demonstrating that binding of DAP12 by siglecs-15 is imperative.^165^

Siglec-15-targeted therapy mediated by its neutralizing effect on antibodies was confirmed to be effective; for example, it inhibited osteoclastic bone resorption, promoted fracture healing, attenuated postmenopausal osteoporosis, and promoted bone formation in various studies.^158,166,167^ Decreased TRAP activity and impaired actin ring formation in mice were observed in the presence of anti-siglec-15 antibodies.^165^ *Siglec-15* siRNA coupled with manganese dioxide nanoparticles was shown to suppress bone metastasis of lung adenocarcinoma cells by inhibiting osteoclast differentiation.^168^ Most importantly, siglec-15 was observed to be exclusively expressed on the surface of osteoclasts but not on CD11b^+^F4/80^+^ macrophages, suggesting that monoclonal antibodies against siglec-15 have great potential for the treatment of arthritis.^158^

Moreover, studies have focused on the role of other siglec molecules in osteoclasts. Siglec-9 is a CD33-related protein that recognizes α (2, 3)-linked sialoglycan structures. Siglec-9 and its murine homolog siglec-E have been shown to inhibit osteoclast activity and bone resorption. While siglec-7 is also expressed on osteoclasts, blocking siglec-7 had no effect on either osteoclast maturation or its function.^169^

### Galectin

Galectin is a relatively conserved class of glycan-binding proteins with a special affinity for β-galactoside. To date, fifteen galectins (galectin-1 to galectin-15) have been identified in mammals. All galectins contain one or more carbohydrate recognition domains (CRDs) that contain approximately 130 amino acids responsible for binding carbohydrates. Galectin is distributed in the nucleus, cytoplasm and cell membrane and can be secreted into the extracellular medium by cells through nonclassical secretory pathways. Galectins play various roles in innate and adaptive immune responses. They are widely involved in many physiological and pathological processes, such as pathogen recognition, inflammatory responses, tumor microenvironment formation and tumor metastasis.^170,171^ The ligand for almost all members of the mammalian galectin family is β1,4-N-acetylglucosamine (LacNAc), a sugar moiety present on almost all cell surfaces.^172^ Galectin family members show different binding affinities for LacNAc: gal-3 binds to the nonreducing end and internal LacNAc; Gal-1 binds to the nonreducing end LacNAc and is eliminated by α2,6 sialylation; and the binding ratio of Gal-10 to β-mannoside increases galectin binding to β-galactoside.^170^ Although LacNAc is the primary ligand for galectins, few studies have linked LacNAc to the effects of galectins on osteoclasts. However, several studies have focused on the effects of LacNAc and galectin analogs on osteoclastogenesis. Galatrox, a C-type lectin isolated from snake venom, shows the ability to bind cell surface glycans. Galatrox binding to LacNAc, especially type II LacNAc (Galβ1-4GlcNAcβ), promoted the secretion of IL-6 and TNF-α by BMM cells, and this effect was through a TLR4-mediated MyD88-dependent signaling pathway.^173^

Galectin-3 (Gal-3) was the first identified and has been the most widely studied.^174^ Gal-3 is expressed in osteoclasts and their precursor cells and can be secreted extracellularly. Its expression can be induced by M-CSF.^175–177^ Gal-3 expression is persistently elevated in the plasma of patients in both the early and chronic stages of RA.^178^ Secreted Gal-3 has two forms: intact secreted Gal-3 and cleaved Gal-3. Myosin-2A inhibits osteoclast precursor fusion and bone resorption capacity.^179^ During osteoclast maturation, intact Gal-3 interacts with myosin 2 A to block the inhibitory effect of Myosin-2A on osteoclastogenesis, manifested by increased RANKL/RANK downstream pathway activation during fusion.^176^ The conversion of intact Gal-3 to cleaved Gal-3 might result in attenuated osteoclast differentiation.^176^ Galectin-3 also mediates its intracellular effects on osteoclast activity by binding to low-density lipoprotein receptor-related protein 1 (Lrp1), thereby increasing RhoA activation, sealing zone formation, and bone resorption.^180^

Adding recombinant human Gal-3 to osteoclasts in culture significantly increased osteoclast production.^178^ Moreover, using the Gal-3 inhibitor GB1107, an siRNA to reduce the expression of endogenous Gal-3, or an extracellular CRD recognition domain that blocks Gal-3 protein can inhibit the formation of osteoclasts.^176,178^ However, some studies have shown that recombinant Gal-3 administered in vitro significantly inhibited the ability of human peripheral blood monocytes, mouse osteoclast precursor cells, and rat bone marrow cells to differentiate into osteoclasts.^181,182^ The volume of bone trabeculae was decreased and the number of osteoclasts was increased in galectin-3 gene knockout mice. However, the number of mature osteoclasts and the bone resorption activity of the osteoclasts decreased.^177,182^ 1α,25-(OH) D acts on VDR and inhibits osteoclast formation by increasing Gal-3 expression.^183,184^

In addition to galectin-3, other members of the galectin family play important roles in regulating osteoclasts. For example, galectin-8 can increase RANKL secretion by activating the ERK signaling pathway in osteoblasts to promote bone loss.^185^ *Gal-1*^*−/−*^ mice showed impaired bone development and loss of bone mass. Bone resorption activity was significantly increased; however, the number of osteoclasts was not affected.^186^ Galectin-9 was identified as a ligand of Tim-3. It generally participates in immune tolerance and tumor immune escape. Galectin-9 has been found in two forms on osteoclasts: as a membrane receptor and a secreted protein. Galectin-9 secreted into the microenvironment promotes T-cell apoptosis by binding to Tim-3 on the surface of T lymphocytes, mediates immune escape of multiple myeloma cells, and aggravates bone injury in patients.^187^ In addition, osteoclasts and their precursors can express Tim-3. Galectin-9 can significantly inhibit the generation of osteoclasts by combining Tim-3 through the Tim-3 CRD on the surface of osteoclasts. The use of recombinant galectin-9 in rats with adjuvant-induced arthritis inhibited inflammatory bone destruction, indicating that exogenous galectin-9 has therapeutic potential in patients with RA.^188^

## Glycosylation changes in bone development and disease

Patients with congenital glycosylation disorders often have skeletal system abnormalities, especially osteoporosis and bone erosion, indicating that glycosylation plays an important role in bone development. For example, patients with phosphoglucomutase 3 (PGM3) deficiency exhibit severe combined immunodeficiency and skeletal dysplasia;^189^ patients with N-acetylgalactosaminyl transferase 3 (GALNT3) deficiency develop severe hyperphosphatemia, accompanied by paroxysmal bone pain, cortical hypertrophy and periosteal reaction;^190^ and genetic mutations in glypican 4 (GPC4), a member of the cell surface heparan sulfate proteoglycan family called Glypicans, can lead to Keipert syndrome, characterized by craniofacial and finger deformities.^191^

Glycosylation of antibodies in patients with arthritis is different from that in healthy people. It is related to disease pathogenesis and inflammation, which is very important for the classification and treatment of diseases.^192–196^ In addition, the proinflammatory function of synovial fibroblasts in rheumatoid arthritis (RA) is related to the remodeling of glycans.^197^ These findings suggest that glycosylation plays an important role in the pathogenesis of joint diseases.

Additionally, abnormal glycosylation is involved in bone metastasis in a variety of tumors and is considered a marker of malignant tumors. Breast cancer is one of the malignant tumors that is most prone to bone metastasis. N-glycans abundance is increased during breast cancer metastasis. High levels of mannose, fucosylation and complex N-glycan can be used as markers and therapeutic targets for bone metastatic breast cancer.^189^ Heparanase cleaves the heparan sulfate chain and is highly expressed in some patients with myeloma. Yang et al. reported that heparanase can promote the homing of myeloma and osteolysis cells by upregulating the expression and secretion of RANKL.^198,199^ Esposito et al. demonstrated that the interaction of Golgi glycoprotein 1 (Glg1) with E-selectin plays a key role in mediating bone metastasis of tumors.^200^

## Conclusion and prospects

### The growing significance of glycoscience in the years ahead

Every living cell in nature produces a complex and varied array of glycans that are essential for the development and survival of biological entities. Glycans are involved in nearly all disease processes affecting humans and other animals. Because they are degradable and metabolized slowly, glycans are able to provide support to tissue cells and act as carriers for the sustained release of drugs.^201^ Gels made of CMT (carboxy methyl tamarind):HEMA (hydroxyethyl methacrylate) can support the growth of osteoclast precursor cells (RAW264.7 cells), neural cells (Neuro2a cells) and human umbilical vein endothelial cells.^202^ CMT:HEMA hydrogels also act as a drug delivery system to release active molecules that promote or inhibit osteoclast formation.^203^ Multifunctional hydrogels prepared via the combination of salmon calcitonin oxidized calcium alginate (SCT-OCA) conjugate and hydroxypropyl chitin (HPCH) extended the half-life of salmon calcitonin and have been used to regulate calcium metabolism and treat osteoporosis.^204^ The implications of delving deeper into the field of glycans are now clear, as their indispensable contributions make clear the mechanisms underlying most diseases and promote the design of effective treatments.

### Technological breakthroughs in unraveling glycan structure and function

Mass spectrometry, nuclear magnetic resonance (NMR), and cryo-electron microscopy (cryo-EM) instrumentation tools and techniques have been markedly advanced in recent years. These advances has led to the expectation that an extremely precise description of the three-dimensional structure of complex sugars is forthcoming. Ultimately, these technologies will be used to reveal the complex interplay between the structure and function of glycoplexes in response to extracellular signals.

Due to their complex stereochemistry and water solubility, the field of glycan synthesis has become one of the most complex areas of study in synthetic organic chemistry. The combination of glycosyltransferases and chemically engineered glycan precursors has become an indispensable approach for precision-driven complex glycan construction. In the future, the synthesis of glycans will become almost as widespread and easy as the synthesis of nucleic acids and proteins.

The comprehensive elucidation of numerous genomes has exerted a profound impact on the progress of glycoscience. The sequencing and classification of genes relevant to glycoscience has facilitated an unparalleled understanding of the evolutionary trajectory and function of genes underlying glycosylation (often referred to as “glycogenes”). These studies hold promise for the discovery of novel glycosylases and for the elucidation of complex mechanisms governing the evolution of distinct glycoforms and glycoforms in different species.

### Future direction and path

Data gathered from glycan profiling, animal models, and clinical relevance convincingly underscore the critical role of cell surface glycans in exacerbating joint inflammation and driving metastatic spread of tumor cells to bone. However, the complex mechanisms underlying the contribution of glycans to these complex processes remain largely unknown.

To date, a comprehensive central repository of various glycan structures is still conspicuously lacking. A global coordinated effort led by prominent countries has led to a consensus on a standardized nomenclature for glycan descriptions in computer databases. This initial progress has laid the foundation for the seamless integration of glycome information into mainstream protein databases.

As our understanding of glycan function continues to advance, glycan modifications are no less important than polypeptide backbones, DNA or RNA. In the long term, glycobiology is destined to be integrated into a holistic approach to the study of biological systems.
